# Research advances of *Zanthoxylum bungeanum Maxim.* polyphenols in inflammatory diseases

**DOI:** 10.3389/fimmu.2024.1305886

**Published:** 2024-01-26

**Authors:** Jinxin Qi, Zhaoping Pan, Xiaoyun Wang, Nan Zhang, Gu He, Xian Jiang

**Affiliations:** ^1^ Department of Dermatology, West China Hospital, Sichuan University, Chengdu, China; ^2^ Laboratory of Dermatology, Clinical Institute of Inflammation and Immunology, Frontiers Science Center for Disease-Related Molecular Network, West China Hospital, Sichuan University, Chengdu, China; ^3^ State Key Laboratory of Southwestern Chinese Medicine Resources, Hospital of Chengdu University of Traditional Chinese Medicine, School of Pharmacy, Chengdu University of Traditional Chinese Medicine, Chengdu, China

**Keywords:** *Zanthoxylum bungeanum Maxim.*, inflammation, polyphenols, inflammatory disease, NF-κB

## Abstract

*Zanthoxylum bungeanum Maxim*., commonly known as Chinese prickly ash, is a well-known spice and traditional Chinese medicine ingredient with a rich history of use in treating inflammatory conditions. This review provides a comprehensive overview of the botanical classification, traditional applications, and anti-inflammatory effects of *Z. bungeanum*, with a specific focus on its polyphenolic components. These polyphenols have exhibited considerable promise, as evidenced by preclinical studies in animal models, suggesting their therapeutic potential in human inflammatory diseases such as ulcerative colitis, arthritis, asthma, chronic obstructive pulmonary disease, cardiovascular disease, and neurodegenerative conditions. This positions them as a promising class of natural compounds with the potential to enhance human well-being. However, further research is necessary to fully elucidate their mechanisms of action and develop safe and effective therapeutic applications.

## 1 Introduction

Chinese prickly ash, also known as Hua Jiao in Mandarin, belongs to the genus *Zanthoxylum* in the Rutaceae family (1). Widely cultivated in Asia, including China, Japan, India, and Korea (2), the genus comprises approximately 250 species, with 41 found in China (**Table 1**) (3). Chinese prickly ash, or Hua Jiao, is a popular spice and traditional Chinese medicine ingredient specifically derived from *Zanthoxylum bungeanum Maxim.* and *Zanthoxylum schinifolium*, according to the Pharmacopoeia of the People’s Republic of China (4). This review, we will focus on *Zanthoxylum bungeanum Maxim. (Z. bungeanum)*.

**Table 1:** Species of the genus *Zanthoxylum* in China.

*Z. acanthopodium*	*Z. collinsiae*	*Z. khasianum*	*Z. molle*	*Z. pilosulum*	*Z. stipitatum*
*Z. ailanthoides*	*Z. dimorphophyllum*	*Z. kwangsiense*	*Z. motuoense*	*Z. pteracanthum*	*Z. tomentellum*
*Z. armatum*	*Z. dissitum*	*Z. laetum*	*Z. multijugum*	*Z. rhombifoliolatum*	*Z. undulatifolium*
*Z. austrosinense*	*Z. echinocarpum*	*Z. leiboicum*	*Z. myriacanthum*	*Z. scandens*	*Z. wutaiense*
*Z. avicennae*	*Z. esquirolii*	*Z. liboense*	*Z. nitidum*	*Z. schinifolium*	*Z. xichouense*
*Z. bungeanum*	*Z. glomeratum*	*Z. macranthum*	*Z. oxyphyllum*	*Z. simulans*	*Z. yuanjiangense*
*Z. calcicola*	*Z. integrifolium*	*Z. micranthum*	*Z. piasezkii*	*Z. stenophyllum*	

*Zanthoxylum bungeanum Maxim.*, commonly known as Honghuajiao, is a deciduous shrub with a height range of 3-7 meters, bearing small, crimson fruits measuring 4-5 mm in diameter. The flowering period spans from April to May, while fruit ripening occurring between August and October. *Z. bungeanum* holds significant importance in both traditional Chinese medicine and cuisine. The earliest record of its use in China can be traced back to the “Book of Songs,” a compilation of folk poetry from the Western Zhou period, underscoring a history of over two thousand years of utilization (5). The dried fruit follicles of *Z. bungeanum* are integral to Chinese cuisine, often incorporated for their distinctive flavor and numbing taste (1). Additionally, leaves at various stages of maturity serve as ingredients and seasonings in Chinese culinary practices (6).

In traditional Chinese medicine, *Z. bungeanum* is esteemed for its properties in warming the spleen and stomach, alleviating pain, and demonstrating anthelmintic and antipruritic effects (4). It is also recognized for promoting the flow of Qi and dispelling coldness (5). Decoctions of *Z. bungeanum* find primary application in treating conditions such as stomachaches accompanied by sensations of coldness and dampness, vomiting, intestinal disorders, diarrhea, ascarid infections, schistosomiasis, and rheumatic joint inflammations (5, 7). Externally, the plant is used to address issues like bruises, eczema, and snakebites (2).

*Z. bungeanum* also features prominently in Indian and Nepalese folk medicine. Its decoction serves as an aromatic tonic for fevers, and as a carminative and stomachic remedy for dyspepsia, cholera, and toothaches (7).

**Figure 1:**
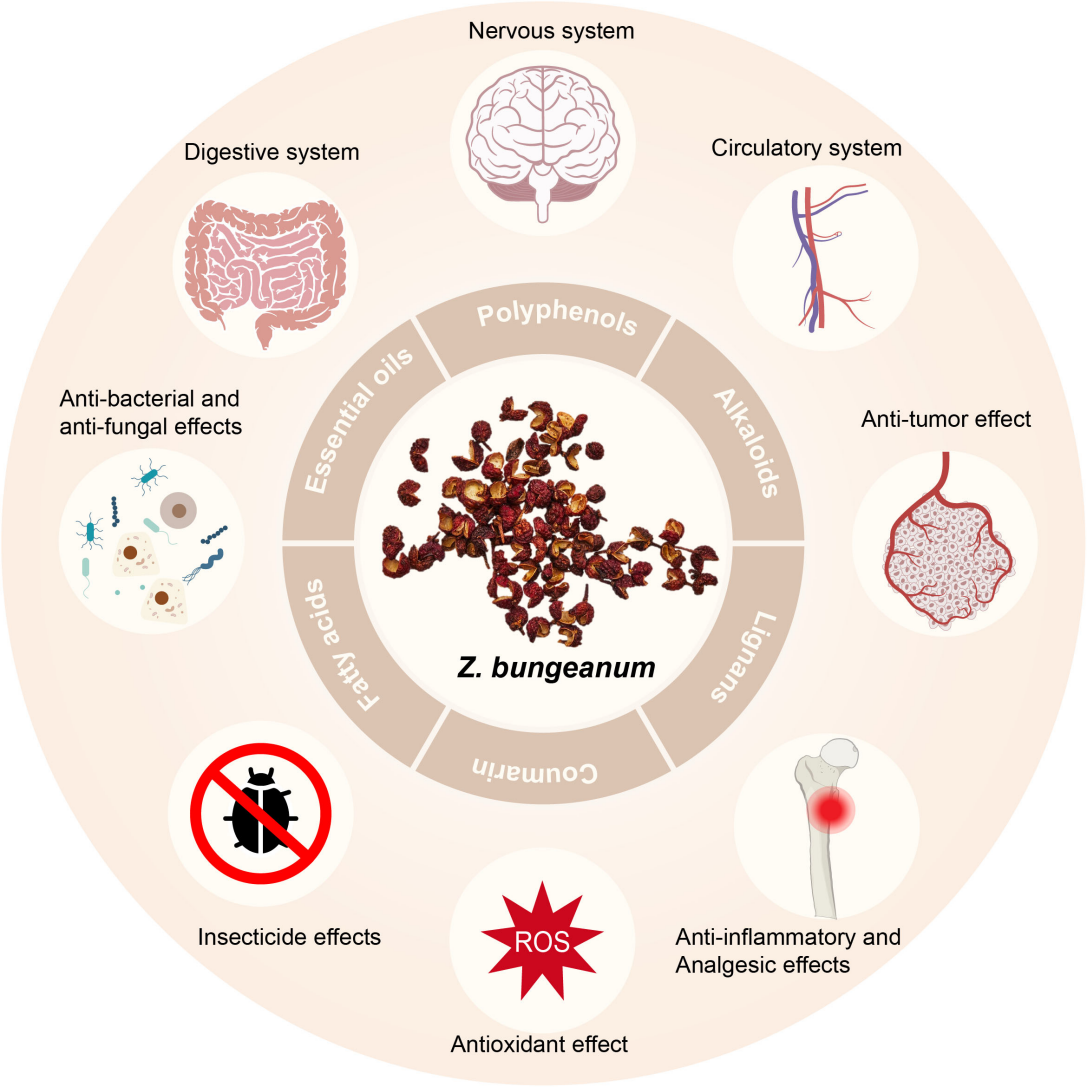
Constituents of *Z. bungeanum* and their pharmacological effects.

Current research endeavors have demonstrated the pharmacological effects of *Z. bungeanum* on the gastrointestinal, neurological, and cardiovascular systems. Additionally, it exhibits anti-inflammatory and analgesic properties, along with displaying antioxidant, anti-tumor, antibacterial, antifungal, and insecticidal effects (2) (**Figure 1**).

Inflammation constitutes an adaptive response of the immune system to deleterious stimuli, encompassing pathogens, cellular injury, and toxic agents. Its principal role is protective, expelling these detrimental agents from the body and instigating the recovery process. However, unbridled inflammation can also be deleterious, culminating in conditions such as atherosclerosis, type 2 diabetes, and rheumatoid arthritis (8). Empirical evidence corroborates the noteworthy anti-inflammatory attributes of polyphenols. They possess the capacity to ameliorate inflammation in various diseases induced by inflammation, such as inflammatory bowel disease and acute pancreatitis (9). The molecular mechanisms underlying the anti-inflammatory activities of polyphenols involve scavenging free radicals, modulating the activity of inflammatory cells, inhibiting enzymes linked to pro-inflammatory attributes like COX2, iNOS, and LOX, suppressing NF-κB and AP-1, and impeding the activation of MAPK, protein kinase C, and Nrf2 (10).

Currently, more than 140 constituents have been identified in *Zanthoxylum bungeanum*, encompassing polyphenols, alkaloids, lignans, coumarin, fatty acids, essential oils, and others (2, 11, 12). Among these, more than 40 polyphenols have been ascertained in *Z. bungeanum*, categorized into various types based on their chemical structures, including flavonoid glycosides, flavonoids, glycosides, phenylpropanoid, anthocyanin and non-glycosides. These polyphenolic compounds have exhibited promising anti-inflammatory effects on disorders affecting diverse organs and systems, comprising ulcerative colitis, arthritis, pain, asthma, UVB-induced skin damage, and cognitive function of the brain ulcerative colitis (13), arthritis (14), pain (15), asthma (16), UVB skin damage (17), and cognitive function of the brain (18). Polyphenols derived from *Z. bungeanum* proficiently inhibit inflammatory cytokines and modulate NF-κB, p38-MAPK, TLR4, Erk1/2, JNK, and Nrf2/HO-1 pathways to exert their anti-inflammatory effects.

In this review, we summarize the polyphenolic compounds present in *Zanthoxylum bungeanum* (*Z. bungeanum*) and the therapeutic effects of *Z. bungeanum* on inflammation, with a particular emphasis on the polyphenols. Recent research suggests that *Z. bungeanum* polyphenols have the potential to significantly contribute to the management and prevention of inflammatory conditions. Further in-depth research is needed to promote their health benefits.

## 2 Composition and structure of polyphenols in *Z. bungeanum*

**Figure 2:**
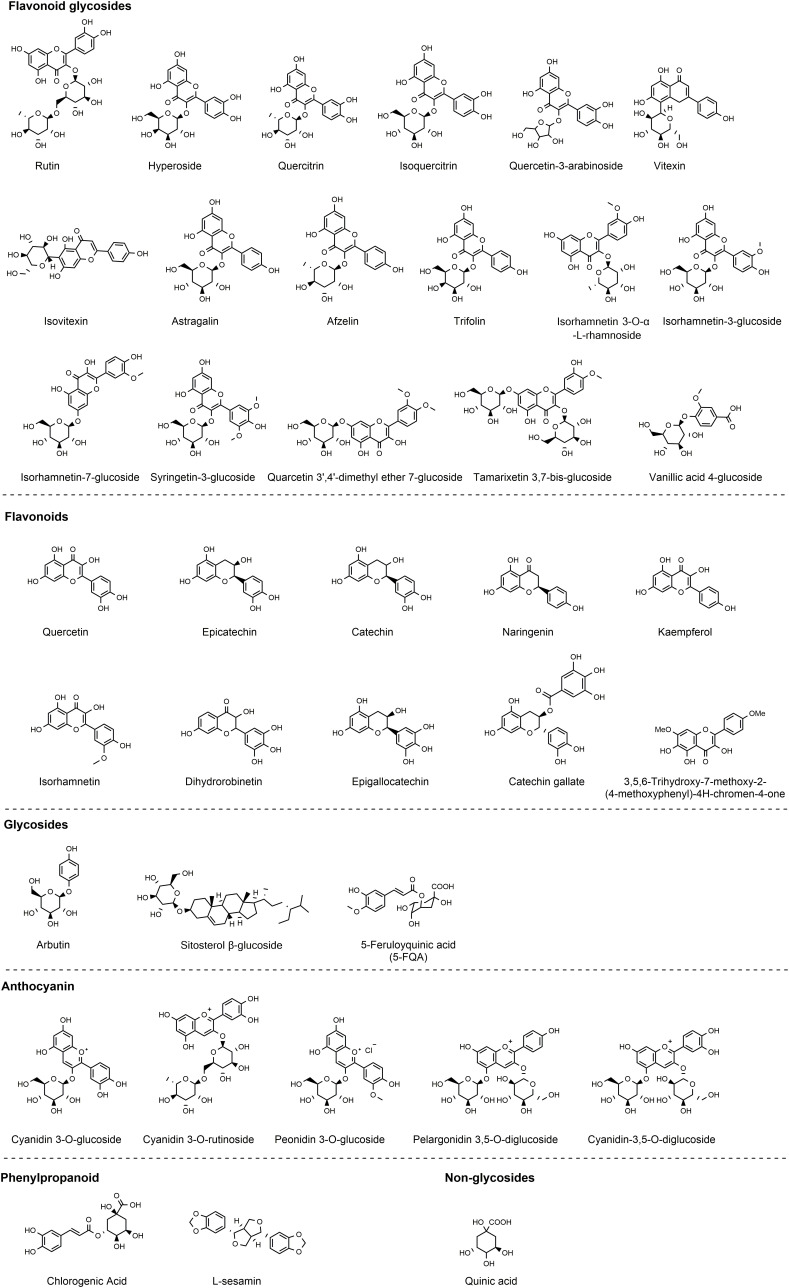
Polyphenols identified in *Z. bungeanum*.

Both the leaves and seeds of *Z. bungeanum* contain polyphenolic compounds, predominantly comprising flavonoid glycosides. Research conducted by three independent groups (19–21) provides substantial evidence of the polyphenol richness in the leaves, characterized by potent antioxidant properties. Noteworthy constituents include 5-feruloyquinic acid, vanillic acid-4-glucoside, quercetin-3-arabinoside, chlorogenic acid, epicatechin, quinic acid, syringetin-3-glucoside, quercetin, isorhamnetin-3-glucoside, trifolin, afzelin, hyperoside, isovitexin, quercitrin, trifolin, rutin, isorhamnetin 3-O-α-L-rhamnoside, astragalin, and isoquercitrin (19–21). In the outer coverings of *Z. bungeanum* fruits, Xiong et al. have identified tamarixetin 3,7-bis-glucoside, quarcetin 3’,4’-dimethyl ether 7-glucoside, 3,5,6-trihydroxy-7,4’-dimethoxyflavone, hyperoside, sitosterol β-glucoside, quercetin, quercitrin, isorhamnetin 7-glucoside, rutin, arbutin, and L-sesamin (22). Additionally, the research conducted by Jia’s group has revealed the presence of epigallocatechin, dihydrorobinetin, naringenin, catechin, kaempferol, catechin gallate, and isorhamnetin are identified by Jia’s group (23). Recently, with the advancement of technology such as the application of high-throughput sequencing techniques, a series of polyphenolic compounds with lower concentrations in *Z. bungeanum* have been identified. The identification of polyphenols in *Z. bungeanum* has expanded from approximately 40 types to over 150 types (24), thanks to these technological developments. Due to words limit, our review specifically revisits polyphenols with higher concentrations in *Z. bungeanum*, focusing on those extensively studied for their anti-inflammatory activities (**Figure 2**).

Investigation of the structure-activity relationships of *Z. bungeanum* polyphenols reveals a correlation between elevated antioxidant efficacy and the presence of a hydroxyl (-OH) group at both the 4’ position on the B ring and the 7 position on the A ring. Moreover, adjacent -OH groups on the B and/or A rings significantly enhanced antioxidant capabilities. Additionally, the diverse structures of these polyphenols suggest that they may display different antioxidant capacities in solution or oil-in-water emulsion reactions (20). *Z. bungeanum* polyphenols have demonstrated effective radical scavenging activities in DPPH, ABTS (21), FRAP, lipid peroxidation inhibition assays (20), and superoxide anion (19). Furthermore, polyphenols have been reported to protect Escherichia coli under peroxide stress (20) and concurrently reduce reactive oxygen species (ROS) levels in HT-29 cells without inducing any cell toxicity (19). Moreover, polyphenols have a cell-protective impact, mitigating oxidative damage in PC12 cells caused by H2O2 (21).

## 3 Inflammatory diseases and polyphenols in *Z. bungeanum*

A combination of polyphenols found in *Zanthoxylum bungeanum* has demonstrated anti-inflammatory effectiveness in both *in vivo* and *in vitro* experiments. The ethyl acetate fraction of *Z. bungeanum* has been identified as the primary active component in enhancing cognitive function in aging mice with D-galactose-induced cognitive decline. This fraction contains several polyphenols, such as hyperoside, chlorogenic acid, quercetin-3β-d-glucoside, rutin, and epicatechin. It aids in reducing neuroinflammation, inhibiting the NLRP3/caspase-1 pathway, GSDMD, and downstream pyroptosis, both in the mouse model and in BV-2 cells subjected to LPS and ATP treatment, leading to overall cognitive improvements (25).

The treatment with *Z. bungeanum* pericarp extract (ZBE), predominantly composed of rutin, isoquercitrin, and quercitrin, has demonstrated effectiveness in protecting mice with dextran sulfate sodium (DSS)-induced ulcerative colitis (UC). It has been observed to mitigate body weight loss, prevent colonic shortening, reduce disease activity index scores, and inhibit myeloperoxidase activity. ZBE is found to inhibit caspase-1, ASC, NLRP3, TLR4, subsequent MAPK and NF-κB pathways, and the production of TNFα, IL-12, and IL-1β, both *in vitro* in the LPS-triggered J774.1 cell model and *in vivo*. Concurrently, activation of PPARγ is detected (13).

In the subsequent section, we will individually discuss the research pertaining to the anti-inflammatory effects of each polyphenolic component found in *Z. bungeanum*. We will categorize the 40 polyphenolic constituents of *Z. bungeanum* into various groups based on their chemical compositions: flavonoids, flavonoid glycosides, glycosides, phenylpropanoid, anthocyanin, and nonglycosides (**Figure 2**). Please note that we do not aim to provide an exhaustive or comprehensive list of all anti-inflammatory studies for each component here. Instead, we have selected those with high citation counts or the most recent research to provide an overview of the association between inflammation and polyphenols in *Z. bungeanum*.

### 3.1 Flavonoid glycosides

#### 3.1.1 Rutin

Rutin is a flavonoid with well-established anti-inflammatory properties (26) Administered at doses of 50-100 mg/kg, rutin exhibits protective effects against hepatotoxicity induced by cyclophosphamide (CP), a potent anticancer agent, in rats. This protection is associated with decreased levels of pro-inflammatory cytokines and signaling molecules, including IL-6, TNFα, iNOS, COX2, p38-MAPK, and NF-κB. Histopathological analysis reveals substantial structural damage to the liver caused by CP, effectively reversed through prior administration of rutin (27). Rutin has also demonstrated the preservation of the vascular barrier integrity in human umbilical vein endothelial cells stimulated by LPS and in an acetic acid-induced mouse mode (28). It effectively reduced hyperpermeability induced by LPS, TNFα, and HMGB1, and suppressed both TNFα production and NF-κB activation triggered by LPS (28). Beyond its anti-inflammatory and vascular protective effects, rutin has demonstrated neuroprotective and anti-colitic properties. In a rat model of spinal cord injury, rutin administration significantly attenuated histological alterations and reduced tissue damage. This was associated with decreased levels of oxidative stress markers, pro-inflammatory cytokines, and caspase-1 (29). In a mouse model of DSS-induced colitis, rutin significantly improved several key indicators of disease severity, including the disease activity score, colon length, and the integrity of goblet cells and colon epithelium. Rutin also reduced the expression of a range of oxidative-inflammatory markers, including IgE, IgM, iNOS, HO-1, and ICAM-1, and restored the balance among effector cells, regulatory cells, and B cells. The study revealed a substantial increase in the activation of the PI3K/Akt/GSK3β/MAPKs/NF-κB and p38/MK2 pathways during DSS-induced colitis in the animal subjects, a condition that rutin treatment effectively mitigated. In silico studies supported the specificity of rutin’s interaction with these pathways (30).

In terms of pharmacokinetics, orally administered rutin is absorbed in the small intestine, transferred to the liver via the bloodstream, and eliminated through bile and the kidneys (31). Major metabolites include sulfates and glucuronides of quercetin (32). In rats, Zhang et al. reported elimination rate half-life, area under the curve, and plasma clearance values of 3.345 minutes, 5750 μg min/ml, and 5.891 mL/min/kg, respectively (33). Intravenous rutin accumulates in the liver, with a significant portion then transferred to the small intestine, and is also detected in the lung post-injection (31). Interactions between rutin and drugs were studied as well. Rutin reduces the anticoagulant effect of racemic warfarin by 31% when co-administered orally. This outcome was ascribed to a noteworthy 77% rise in the unbound formation clearance of both oxidative and reductive metabolites, coupled with an elevation in the unbound renal clearance of the more potent S-enantiomer of warfarin (34). Rutin also significantly decreases the oral Cmax and AUC of cyclosporine by 63.2% and 57.2%, respectively, through the activation of Pgp transporter and CYP3A enzyme (35).

#### 3.1.2 Hyperoside

Hyperoside is another flavonoid known for its anti-inflammatory properties. In mouse peritoneal macrophages subjected to LPS stimulation, hyperoside inhibited TNFα, IL-6, and NO production by 32.3%, 41.3%, and 30%, respectively. Moreover, hyperoside reduced NF-κB activation and IκB-α degradation (36). This compound also exhibits anti-neuroinflammation effect *in vitro* and *in vivo* (37, 38). In the LPS-induced HT22 murine neuronal cell line, hyperoside enhances cell survival and mitigates inflammation, oxidative stress, and apoptosis. This effect is achieved by amplifying SIRT1, triggering the activation of both Wnt/β-catenin and sonic hedgehog pathways (38). In rats, 50 mg/kg hyperoside protected against cerebral ischemia-reperfusion injury by mitigating oxidative stress, inflammation, and cell death. Rats treated with hyperoside exhibited significantly enhanced neurological function and a substantial reduction in the ratio of cerebral infarction volume (37).

Hyperoside also attenuate several vascular inflammatory responses initiated by elevated glucose levels in human umbilical vein endothelial cells and mice. These responses include vascular permeability, monocyte attachment, CAMs expression, ROS formation, and NF-κB activation (39). Furthermore, hyperoside’s anti-arthritic properties have also been verified both *in vitro* and *in vivo*. It can suppress inflammation and prevent cartilage breakdown by influencing the PI3K/AKT/NF-κB and MAPK signaling pathways, as well as the interplay between the Nrf2/HO-1 and NF-κB signaling pathways (40). Hyperoside also inhibited OVA-induced airway hyperresponsiveness in mice through activation of Nrf2/HO-1 (41). In a rat model of antiphospholipid syndrome (APS), hyperoside at a dose of 40 mg/kg led to increased fetal weight, reduction of fetal resorption rates, and reduced pregnancy loss by modulating the mTOR/S6K and TLR4/MyD88/NF-kB signaling pathways (42).

#### 3.1.3 Quercitrin

Quercitrin demonstrates the ability to attenuate carbon tetrachloride (CCl4) induced brain injury by suppressing ROS, MDA, TNFα, and IL-6 (43). Furthermore, it exhibits protective effects against skin damage induced by UVB damage. This protection is achieved through the reduction of ROS, NF-κB activation, and DNA damage triggered by UVB exposure. Quercitrin also restores the diminished expression of catalase and the GSH/GSSG ratio due to UVB exposure (17). In a study involving mice with Alzheimer’s disease, quercitrin inhibits the activation and proliferation of microglia, decreases the accumulation of amyloid-β plaques, and improves cognitive impairment by inhibiting inflammation. Specifically, this compound inhibits the level of IL-1α, IL-17A, IL-6, and G-CSF in peripheral blood, as well as IL-1α, IL-4, IL-6, Eotaxin, CXCL-1, MIP-1α, MIP-1β and G-CSF in the brain, thereby alleviating systemic inflammation in the 5XFAD mice (44).

Quercetin and quercitrin, common flavonoids in vegetables, are frequently compared (45). Theoretical calculations clarify that the oxygen atom located on the B rings could serve as the primary site for alterations in electron cloud density, providing insights into how quercetin and quercitrin exert their anti-inflammatory and ROS scavenging effects (46). In LPS-stimulated RAW264.7 cells, both compounds markedly decrease NO and ROS production, as well as the expression of TNFα, IL-1β, and IL-6 (46). However, Comalada et al. reported that unlike quercitrin, quercetin can reduce the expression of cytokines and iNOS by inhibiting the NF-κB pathway *in vitro* in bone marrow-derived macrophages, without affecting c-Jun N-terminal kinase activity. The group revealed that quercitrin’s *in vivo* impact in a rat colitis model induced by DSS may be attributed to the liberation of quercetin, which occurs following the breakdown of glycosides by intestinal microbiota. In other words, quercitrin releases quercetin to exert its anti-inflammatory influence, achieved by inhibiting the NF-κB pathway (45).

#### 3.1.4 Isoquercitrin

Isoquercitrin has undergone tested in an LPS-stimulated RAW264.7 cell model, revealing its ability to decrease NO production, downregulate the expression of PGE2, COX2, iNOS, and NF-κB p65 protein, and reduce the mRNA levels of IL-1, IL-6, PTGES2, and MCP-1 (47). Moreover, at a dosage of 20 mg/kg, isoquercitrin has demonstrated the capacity to protect denervated muscle from atrophy. This protective effect is achieved by reducing the levels of IL-1β, TNFα, and IL-6 and inactivating the JAK/STAT3 signaling pathway in the target muscle (48).

#### 3.1.5 Vitexin

Vitexin exhibits anti-inflammatory properties in the OVA-induced mouse allergic asthma model at doses of ranging from 0.2 to 5 mg/kg. Specifically, vitexin mitigates the migration of eosinophils, neutrophils, and mononuclear cells prompted by OVA within bronchoalveolar lavage fluid (BALF). Examination of lung tissue reveals that vitexin effectively suppresses the invasion of leukocytes, mucus production, and development of pulmonary edema. It also moderates the escalation of Th2 cytokines in BALF and reduces the concentration of IgE in the plasma (49). Vitexin has also demonstrated anti-inflammatory effects in chronic cerebral hypoperfusion injury in a rat model of persistent bilateral common carotid artery occlusion and in HT22 mouse hippocampal neuronal cells exposed to oxygen and glucose deprivation followed by reoxygenation injury. The findings confirm vitexin’s ability to modulate Epac and NLRP3. Additionally, in the rat model, vitexin has shown the potential in diminishing the severity of ongoing pathological harm in the cortex and hippocampus and preventing further decline in cognitive function (18). Moreover, vitexin inhibits inflammatory pain in various mouse models of inflammation-related pain, including acetic acid-induced writhing, pain-like behavior prompted by phenyl-p-benzoquinone, capsaicin, complete Freund’s adjuvant (CFA), and both phases of the formalin test. It also alleviates mechanical and thermal hyperalgesia triggered by capsaicin, carrageenan, and chronic CF. TRPV1 is considered the key target (50). Additionally, vitexin alleviates liver inflammation in a DSS-induced colitis model by inhibiting the TLR4/NF-κB signaling pathway activation. Administration of vitexin results in lower ALT and TC levels in the livers of mice suffering from liver injury. It also reduces the release of IL-6, TNFα, and IL-1β induced by DSS (51). Furthermore, vitexin inhibits the movement of neutrophils toward areas of inflammation by suppressing the p38, ERK1/2, and JNK pathways (52).

#### 3.1.6 Isovitexin

Isovitexin effectively alleviates contact dermatitis in mice triggered by ginkgolic acids, leading to a significant reduction in ear swelling, splenomegaly, and inflammatory cell infiltration. Subsequent investigations have revealed that isovitexin can impede the MAPK and STAT signaling pathways, along with the phosphorylation of SHP2 (53). In the mouse models of kidney injury induced by cyclophosphamide (CP) (54), liver injury triggered by LPS/d-galactosamine (55), and acute lung injury induced by LPS (56), isovitexin demonstrates its therapeutic effects via inhibiting NF-κB activation and inducing Nrf2 and HO-1 expression. In the kidney injury model, isovitexin mitigates CP-induced increases in serum BUN and creatinine, and curbs TNFα, IL-1β, and IL-6 (54). Isovitexin substantially diminishes liver injury, evidenced by reduced histopathological changes and lower AST and ALT levels. It also reduces TNFα levels, MPO activity, and MDA content (55). Pretreatment with isovitexin significantly alleviates acute lung injury, as demonstrated by reduced histopathological changes, diminished granulocyte infiltration, and subdued endothelial activation. Additionally, it lowers VCAM-1 and ICAM-1 expression, reduces MPO and MDA levels, and enhances GSH and SOD (56).

#### 3.1.7 Astragalin

Astragalin notably alleviates inflammatory reactions and bone damage in both DBA/1J mice with collagen-induced arthritis and human fibroblast-like synoviocytes. It reduces joint swelling, arthritis index, and bone erosion, while also inhibiting the production of IL-1β, TNFα, IL-6, and IL-8. Moreover, a decrease in MMP-1, MMP-3, and MMP-13 levels has also been observed in chondrocytes, synovial cells, and TNFα-induced MH7A cells. Additionally, astragalin inhibits p38, JNK phosphorylation, and c-Jun/AP-1 activation (57). Furthermore, through the ROS and MAPK signaling pathway, the process of osteoclastogenesis in inflammatory osteolysis is alleviated by astragalin (58). In an OVA-challenged mouse model, astragalin at doses of 10-20 mg/kg impedes mast cell recruitment, preventing airway thickening and alveolar emphysema (59).

#### 3.1.8 Afzelin

Afzelin performs anti-inflammatory effect in two *in vitro* experiments (60, 61). In human keratinocytes exposed to particulate matter (PM), a widespread airborne contaminant, afzelin mitigates inflammation and ROS production. It also inhibits p38 kinase, as well as the transcription factors c-Fos and c-Jun (61). The inhibitory effect of afzelin on the p38 kinase pathway contributes to its protective effect of human keratinocytes and epidermal equivalent models exposed to UVB, resulting in a reduction of IL-6, TNFα, and PGE2 release induced by UVB (60).

### 3.2 Flavones

#### 3.2.1 Quercetin

Quercetin stands out as one of the extensively researched polyphenols in *Z. bungeanum*. showcasing therapeutic potential in addressing inflammatory conditions, particularly arthritis (62, 63). In a study involving women with rheumatoid arthritis, a daily supplement of 500mg quercetin over 8 weeks resulted in significant improvements in the clinical symptoms, disease activity, hs-TNFα levels, and health assessment questionnaire outcomes (62). For rabbits with surgically-induced osteoarthritis (OA), a 4-week gavage treatment of 25 mg/kg quercetin demonstrated increased SOD and TIMP-1 expressions, reduced MMP-13 expression, and mitigation of OA degeneration, comparable to the effects observed in the celecoxib-treated group (63). Quercetin’s impact extends to inflammation-based pain models, as intraperitoneal and oral administrations significantly suppressed pain induced by phenyl-p-benzoquinone and acetic acid. It also mitigated the second phase of pain intensity escalation caused by formalin and carrageenin. This compound further demonstrated its efficacy in curtailing hypernociception stimulated by TNFα and CXCL1, along with reducing carrageenin-induced IL-1β production (15). Moreover, in RAW264.7 cells stimulated with LPS, quercetin significantly reduced the production of NO, inducible NO synthase, and IL-6. It also hindered the relocation of NF-κB to the cell nucleus and suppressed the activation of Erk1/2 and JNK. In DNCB-induced atopic dermatitis mouse model, quercetin exhibited anti-inflammatory effects, as evidenced by improvements in ear thickness, serum IgE levels, and histological analysis (64).

Regarding the pharmacokinetic aspects of quercetin, initial metabolism occurs in the small intestine through processes like glucuronidation and O-methylation. The subsequent breakdown and processing take place in the liver after reaching it through the hepatic portal vein. Notably, gut bacteria, especially clostridium orbiscindens, play a role in the breakdown process in the large intestine. Key metabolites found in human plasma include quercetin-3-glucuronide, quercetin-3-sulfate, and isorhamnetin-3-glucosidic acid. Quercetin distribution involves various organs (lungs, kidneys, heart, and liver), with the lungs exhibiting the highest concentrations. Conjugates are predominantly present in the blood and are excreted in urine (65).

Pharmacokinetic and pharmacodynamic interactions between quercetin and drugs have been unveiled in studies. Competitive binding to serum albumin influence on cytochrome P450, glycoproteins, and other factors modify drug profiles, affecting treatment outcomes for infectious diseases, cardiovascular diseases, diabetes, and cancer (65). For example, quercetin competes with erlotinib for binding to bovine serum albumin, potentially contributing to increased adverse events associated with erlotinib use (66). Additionally, combined treatment with quercetin and methotrexate significantly reduces inflammatory mediators in collagen-induced arthritis mice, suggesting quercetin’s potential as an adjuvant to enhance anti-rheumatic monotherapy (67).

#### 3.2.2 Epicatechin

Epicatechin exhibits dose-dependent reduction in TNFα-induced increase of JNK, p38, and ERK1/2 phosphorylation, nuclear AP-1-DNA interaction, activation of the NF-κB signaling pathway, nuclear NF-κB-DNA binding, p65 nuclear translocation, and PPARγ expression in 3T3-L1 adipocytes (68). A dosage of 20 mg/kg epicatechin proves effective in mitigating inflammation in the renal cortex of fructose-fed rats (69), while a higher dose of 80 mg/kg demonstrates efficacy in alleviating LPS-induced renal inflammation in rats (70). In both studies, downregulation of TNFα, iNOS and IL-6 are observed (69, 70). Furthermore, a dosage of 15 mg/kg epicatechin exhibits anti-inflammatory properties in mice experiencing LPS-induced acute lung injury, achieved by directly impeding the function of the p38-MAPK signaling pathway (71). Epicatechin also shows significant effects in mitigating atherosclerosis, specifically reducing severe lesions by 27% in ApoE*3-Leiden mice, without affecting plasma lipids. Additionally, it successfully countered diet-induced increases in inflammatory markers such as serum amyloid A and human C-reactive protein (72).

Concerning the pharmacokinetic parameters, orally administered epicatechin is initially absorbed in the duodenum, with the majority (70%) being absorbed in the lower intestine after catabolism by the gut microbiome. Over 80% of ingested epicatechin is absorbed, and the gut microbiome plays a crucial role in its metabolism, yielding more than 20 identifiable metabolites. These metabolites are then mainly excreted through urine (73).

#### 3.2.3 Catechin

Catechin mitigates coronary heart disease in rats induced by pituitrin injection and a high-fat diet by inhibiting, lipoprotein-associated phospholipase A2, C-reactive protein, TNFα, and IL-6. Simultaneously, catechin treatment also demonstrates the inhibition of NF-κB and upregulation of FXR, p-STAT3, and p-Akt expression levels (74). High fructose consumption over a six-week period in rats induces a series of metabolic problems, including insulin resistance, dyslipidemia, obesity, reduced plasma adiponectin, and inflammation of adipose tissue. Supplementing their diet with 20 mg/kg/day of catechin effectively enhances all these parameters. In the TNFα induced 3T3-L1 adipocyte model, catechin inhibits inflammation by suppressing MAPKs, JNK and p38 activation, and preventing PPAR-γ reduction (75). At a dose of 75-300 mg/kg, catechin alleviates allergic symptoms such as sneezing and nose rubbing in mice suffering from OVA-induced allergic rhinitis. It reduces the levels of ovalbumin-specific IgE, IL-5, IL-13, restoring the balance between Th2 and Th1 cells. The potential mechanism of action involves the inhibition of TSLP expression in epithelial cells through the modulation of the NF-κB/TSLP pathway by catechin (76).

#### 3.2.4 Naringenin

Naringenin significantly inhibits paw swelling and pathological changes in the joint tissue in the SD rat model of complete Freund’s adjuvant-induced arthritis. Additionally, IL-1β, TNFα, and IL-6 in serum are notably suppressed (14). Naringenin demonstrates neuroprotective effects by ameliorating neuroinflammation through the inhibition of p38-MAPK and STAT-1. In neuroglial cells induced by LPS/IFN-γ, this compound reduces the production of TNFα and NO, along with the expression of iNOS, thereby preventing neuron death induced by inflammation (77). Furthermore, naringenin inhibits pain behavior in mice triggered by various inflammatory stimuli, including acute pain caused by the use of acetic acid, PBQ, formalin, capsaicin, and CFA, as well as the provocation of mechanical hyperalgesia through subplantar injection of capsaicin, CFA, carrageenan, or PGE2. The mechanism of naringenin involves the activation of NF-κB and the inhibition of IL-1β, IL-33, TNFα, and oxidative stress. Additionally, naringenin activates the analgesic NO-cyclic GMP-PKG-ATP sensitive K+ channel pathway (78). Naringenin also exhibits anti-inflammatory effects in respiratory inflammation. In a murine COPD model, characterized by 90 days of cigarette smoke exposure-induced initiation, 20-80mg/kg of naringenin significantly improves pulmonary function, reduced inflammatory cells, and inhibits IL-8, TNFα, and MMP-9 in mouse BALF and serum. Suppression of the NF-κB pathway is also observed in mice treated with naringenin (79).

Delving into the pharmacokinetic characteristics, orally administrated naringenin exhibits limited absorption in the human gastrointestinal tract, yielding a modest 15% oral bioavailability. The absorption process encompasses both passive diffusion and active transport mechanisms. Once absorbed, naringenin swiftly distribute to vital organs such as the liver, cerebrum, kidney, spleen, and heart, suggesting potential neuroprotection within the central nervous system. Remarkably, naringenin demonstrates high permeability across blood-brain barrier models. The enterohepatic recycling of naringenin plays a crucial role, contributing to hepatic conjugate excretion in bile and participating in the enteric excretion of phase II conjugation. Post-absorption, Naringenin undergoes a significant metabolic process involving glucuronidation, resulting in the detection of 98% of naringenin−o−β−d−glucuronide in plasma. Before absorption in the caecum, naringenin undergoes hydrolysis by beta–glucosidase in the small intestine. Further metabolism by intestinal bacterial microflora produces p−hydroxybenzoic acid, p−hydroxyphenylpropionic acid, and p−coumaric acid, which manifest in plasma and urine. Ultimately, flavonoid excretion primarily occurs through two pathways: the biliary and urinary pathways (80).

#### 3.2.5 Kaempferol

In OVA challenged asthmatic mouse models, oral intake of kaempferol mitigated the increase in eosinophil major basic protein and eotaxin-1 expression, achieve through the transactivation inhibition of NF-κB. Consequently, this reduction leads to decreased accumulation of eosinophils in the airways and lung tissue (16). Furthermore, kaempferol demonstrates the ability to control vascular inflammation in an atherosclerosis rabbit model with a high-cholesterol diet for ten weeks. Following treatment with kaempferol, decreased levels of IL-1β, TNFα, and MDA, an increase in serum SOD activity, and a reduction in the gene and protein expression of aortic E-selectin, ICAM-1, VCAM-1, and MCP-1 are observed (81). In a rat model simulating cerebral ischemia/reperfusion by occluding the middle cerebral artery for 60 minutes and then reperfusion, kaempferol is administered at doses of 25-100 mg/kg. The treatment significantly reduces the volume of cerebral infarction following cerebral ischemia-reperfusion, alleviated inflammation, and prevented the breakdown of the blood-brain barrier, thereby improving the neurological outcome on the 7th day after cerebral ischemia reperfusion. Additionally, reduced nuclear translocation and phosphorylation of the transcription factor NF-κB p65 are observed (82). What’s more, kaempferol exerts a protective effect on osteoarthritis chondrocytes by regulating the XIST/miR-130a/STAT3 axis, thereby inhibiting inflammation and extracellular matrix degradation (83).

Limited absorption and minimal oral bioavailability are observed with kaempferol. Its lipophilic nature allows for passive absorption, diffusion facilitation, and active transport. Metabolism in the liver results in the formation of glucuronic acid and sulfate conjugates, while intestinal enzymes in the small intestine contribute to its processing. Aglycogens, produced through the metabolism of kaempferol by colonic microbiota, are further transformed into 4-hydroxyphenylacetic acid, 4-methylphenol, and phloroglucinol. These metabolites undergo absorption into the systemic circulation, distribution to tissues, and eventual excretion in feces or urine (84).

Notably, the administration of a 12 mg/kg kaempferol dose demonstrated a substantial improvement in oral etoposide bioavailability in rats, showing a 64% enhancement compared to lower doses of 47% and 15%. At the highest dose, 12 mg/kg kaempferol exhibited a 26% increase in intravenous etoposide bioavailability. This intriguing finding suggests potential hepatic CYP3A4 inhibition and implicates kaempferol in reducing the unpredictable oral bioavailability of etoposide (85).

#### 3.2.6 Isorhamnetin

Isorhamnetin possesses the ability to inhibit inflammation and provide renal protection. In a rat model of type 2 diabetes induced by a high-fat diet and streptozotocin, isorhamnetin significantly improved the renal function. The study reported that Isorhamnetin inhibited NF-κB signaling activity, resulting in reductions in IL-1β, IL-6, TNFα, TGF-β1, and ICAM-1 levels, as well as the mitigation of oxidative stress in diabetic rats and glomerular mesangial cells (86). Research conducted by Dou’s team demonstrated that isorhamnetin exerts beneficial effects on TNBS- and DSS-induced mouse inflammatory bowel disease (IBD) by upregulating xenobiotic metabolism mediated by PXR and concomitantly downregulating NF-κB signaling. Isorhamnetin inhibited the expression of IL-6 and TNFα, as well as the mRNA levels of ICAM-1, iNOS, TNFα, COX2, IL-6, IL-2, through the aforementioned pathways (87). Isorhamnetin has been found to inhibit neuroinflammation. In BV2 microglial cells stimulated with LPS, isorhamnetin significantly inhibits NO and PEG2, as well as IL-1β, TNFα, iNOS and COX2. Research on its anti-inflammatory mechanism indicates that isorhamnetin controls neuroinflammation by inhibiting the TLR4/MyD88/NF-kB pathway (88). Moreover, isorhamnetin exhibits efficacy in asthma. In TNFα-induced human bronchial epithelial cell line BEAS-2B, isorhamnetin at concentrations of 20-40 μM can reduce cellular proliferation and notably suppress the expression of CXCL10, IL-1β, IL-6, and IL-8. Furthermore, treatment with isorhamnetin downregulates the phosphorylation of the NF-κB and MAPK pathways in this model (89).

In the context of collagen-induced arthritis, isorhamnetin at doses ranging from 10 to 20 mg/kg significantly alleviate arthritis, improving arthritis score, joint damage score, and inflammation score. Isorhamnetin can also regulate the production of cytokines such as IL-1β, TNFα, IL-6, IL-10, IL-17A, IL-17F, and IL-35, while mitigating oxidative stress (90).

### 3.3 Glycosides

Arbutin significantly enhances kidney function in rats experiencing LPS-induced acute kidney damage. It reduces inflammation and cell death by modulating the PI3K/Akt/Nrf2 pathway after LPS exposure both *in vivo* and *in vitro*. Moreover, the Akt inhibitor GDC effectively inhibits this arbutin-induced improvement *in vitro* (91). Additionally, arbutin protects mice from isoproterenol (ISO)-induced cardiac hypertrophy. Pre-treatment with arbutin notably inhibits the TLR4/NF-κB pathway, resulting in decreased IL-6 and TNFα (92). In a DSS-induced mouse colitis model, arbutin significantly mitigates symptoms such as elevated disease activity index, loss of body weight, and increased colon weight-to-length ratio. This anti-inflammatory impact is contingent upon the control of JAK2 and the suppression of IL-1β, TNFα, and IL-6. Arbutin also suppresses inflammatory responses in epithelial (IEC6) and immune (RAW264.7) cells triggered by LPS. However, these benefits, both *in vitro* and *in vivo*, can be negated by the JAK2 inhibitor AG490 (93). In addressing metabolic issues, arbutin is found to suppress high-glucose-induced inflammation in adult human retinal pigment epithelial cells via upregulation of SIRT1, which provides a novel therapeutic target for diabetic retinopathy management (94). In arbutin-treated LPS-triggered BV2 murine microglial cells, inhibition of NO production, and reduced expression of COX2 and iNOS are observed. Arbutin significantly diminishes the expression of IL-1β, IL-6, MCP-1, and TNFα. Additionally, it impedes the nuclear transcriptional and translocation activity of NF-κB (95).

Jin’s group developed arbutin-loaded gelatine methacryloyl-Liposome microspheres (GM-Lipo@ARB), offering extended arbutin release and notable cartilage targeting. The microspheres decrease inflammation in IL-1β-stimulated arthritic chondrocytes and maintain cartilage matrix equilibrium through NF-κB inhibition and Nrf2 pathway activation. Application of the GM-Lipo@ARB lessens inflammation and oxidative stress in articular cartilage, effectively decelerating osteoarthritis progression in a mouse model (96).

### 3.4 Phenylpropanoid

The anti-inflammatory effects of chlorogenic acid (CGA) have been investigated in LPS-stimulated RAW 264.7 macrophages and BV2 microglial cells. CGA inhibits the production of NO, IL-1β, IL-6, TNFα, CXCL1, COX2, and iNOS. A possible mechanism of action involves the reduction of ninjurin1 level and nuclear translocation of NF-κB (97). CGA also downregulates the TLR4/MyD88/NF-κB signaling pathway (98, 99). Through this pathway, CGA can potently inhibit CCl4-induced liver fibrosis in rats (98), and alleviate renal inflammation in a mouse model of hyperuricemia induced by hypoxanthine and potassium oxonate (99). Animal experiments have confirmed that the systemic administration of CGA can help alleviate both inflammatory and neuropathic pain (100).

The hydrophilic nature of CGA essentially impedes its passage through the lipophilic membrane barrier, resulting in low absorption. Absorption likely occurs in the stomach rather than the small intestine. Caffeic acid is detected in plasma and urine 1.5 hours after a CGA-supplemented meal, along with derivatives like ferulic acid and isoferulic acid. These derivatives result from CGA hydrolysis in the small intestinal mucosa. CGAs’ absorption and metabolism are relatively low, constituting about one-third of total intake in the upper gastrointestinal tract. The remaining two-thirds reach the colon, where intense microbial metabolism occurs. Microflora-derived esterase hydrolyzes CGA, producing microbial metabolites, comprising 57.4% of the total CGA consumed, emphasizing the crucial role of gut microbiota in CGA metabolism and biological properties (101).

### 3.5 Anthocyanin

Anthocyanins, a member of the polyphenolic family in *Z. bungeanum*, contribute to the crimson coloration of its fruit peel. (102). In total, five types of anthocyanins with clear chemical structure have been identified in *Z. bungeanum* (24, 102–104).

The anti-inflammatory efficacy of cyanidin 3-O-glucoside (C3G) has been demonstrated across various *in vivo* and *in vitro* models. C3G exhibits the ability to safeguard mice from chronic skin damage induced by UVB exposure, leading to notable improvements in UVB-induced epidermal hyperplasia, collagen fiber preservation, ROS levels, and the expression of COX-2 and IL-6 (105). Furthermore, C3G demonstrates protective effects in rats against cecal ligation and puncture (CLP)-induced acute lung injury (ALI), enhancing their survival rate. C3G treatment results in reduced serum levels of TNF-α, IL-1β, and IL-6, along with the inhibition of COX-2 protein expression and PGE2 production in the lung, potentially through the suppression of the NF-κB signaling pathway (106). C3G also exerts anti-neuroinflammatory effects. In LPS-stimulated BV2 microglia, C3G effectively suppresses microglial activation and the levels of neurotoxic mediators and pro-inflammatory cytokines. Moreover, there is observed suppression of the NF-κB and p38 MAPK signaling pathways (107). Additionally, in TNBS-challenged mice, C3G significantly ameliorates clinical symptoms and mitigates histological damage, possibly by protecting the intestinal barrier and suppressing inflammatory cytokine secretion (108).

Cyanidin 3-O-rutinoside, peonidin 3-O-glucoside, pelargonidin 3,5-O-diglucoside, cyanidin-3,5-O-diglucoside have limited study in inflammatory disorders. Only a few *in vitro* studies were found (109, 110).

### 3.6 Non-glycosides

Research on the anti-inflammatory effects of quinic acid is limited. However, one study shown that quinic acid mitigates vascular inflammation in TNFα-stimulated vascular smooth muscle cells by reducing MAPK phosphorylation and inhibiting NF-κB activation (111).

Limited study has been conducted on the anti-inflammatory effects of catechin gallate, epigallocatechin, dihydrorobinetin, quercetin-3-arabinoside, quarcetin 3’,4’-dimethyl ether 7-glucoside, isorhamnetin-3-glucoside, isorhamnetin 7-glucoside, isorhamnetin 3-O-α-L-rhamnoside, tamarixetin 3,7-bis-glucoside, 3,5,6-trihydroxy-7,4’-dimethoxy flavone, sitosterol β-glucoside, trifolin, vanillic acid-4-glucoside, syringetin-3-glucoside, L-sesamin, and 5-feruloyquinic acid.

## 4 Direct target of *Z. bungeanum* polyphenols

**Figure 3:**
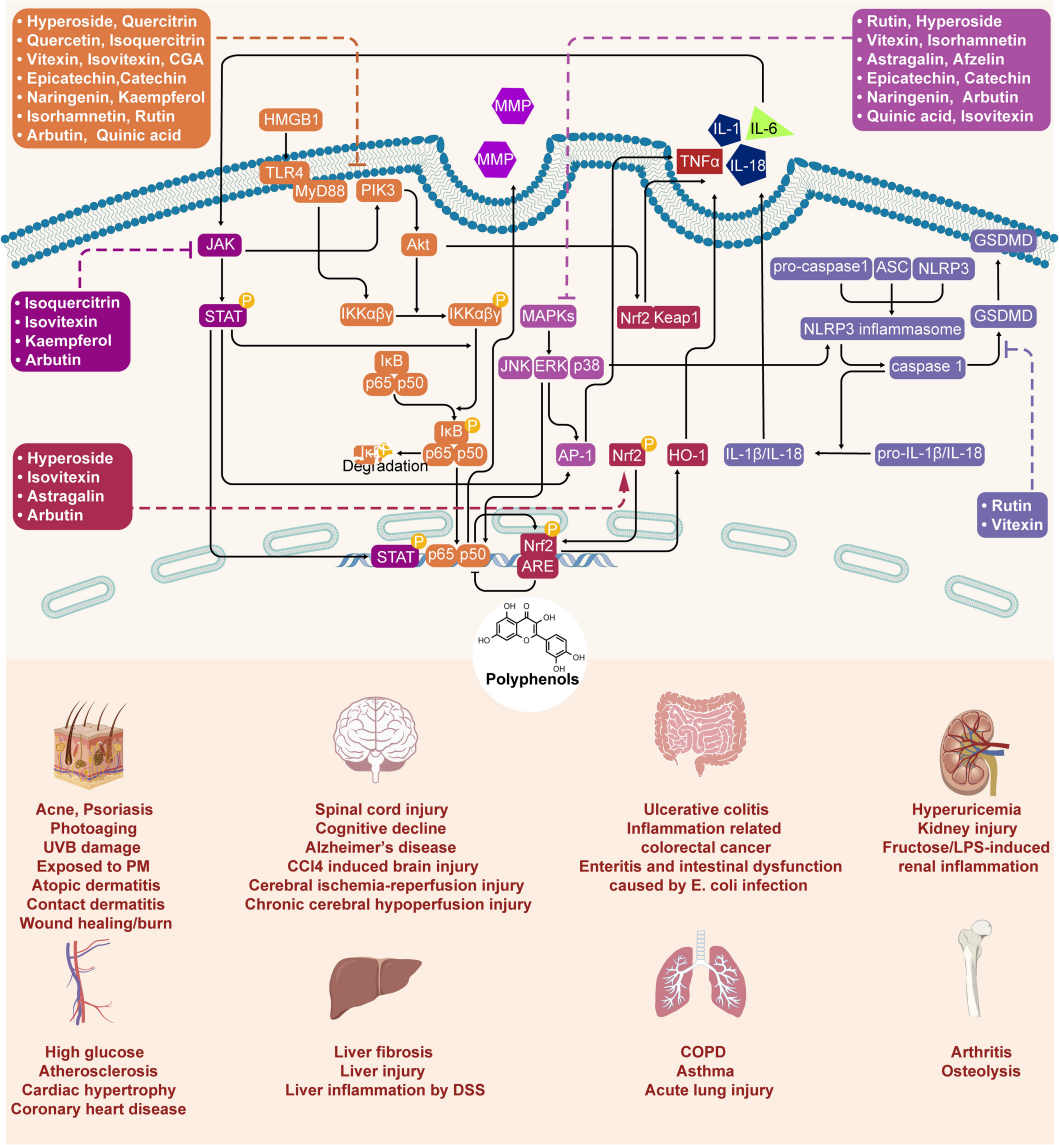
Molecule mechanism of polyphenols in *Z. bungeanum* and their anti-inflammatory effect.

In the preceding sections, we primarily delineated the anti-inflammatory pharmacological activities of *Z. bungeanum* polyphenols, highlighting their modulation of inflammation through signaling pathways, including NF-κB, MAPK, Nrf2/keap1, and the NLRP3 inflammasome (**Figure 3**). However, to date, limited research has been conducted on the direct targeting of proteins or genes associated with inflammation by *Z. bungeanum* polyphenols. In this section, we consolidate and summarize the pertinent studies investigating the direct interactions of *Z. bungeanum* polyphenols with inflammatory-related proteins or genes (**Table 2**).

**Table 2:** Direct target of *Z. bungeanum* polyphenols.

Polyphenols	Target	Verified by	Publication
Rutin	HMGB1	SPR	(112)
Quercetin	HMGB1	SPR	(112)
	PI3K1R	SPR	(113)
Kaempferol	HMGB1	SPR	(114)
	TNF- α	SPR	(115)
	CASP3 PARP1	SPR	(116)
	JAK3	IP	(117)

*SPR, Surface plasmon resonance;

BLI, Bio-Layer Interferometry;

IP, Immunoprecipitation;

ChIP, Chromatin immunoprecipitation.

## 5 Clinical trials of *Z. bungeanum* polyphenols

Currently, several clinical trials have utilized *Z. bungeanum* polyphenols; however, their application in inflammatory conditions remains limited. **Table 3** below summarizes completed clinical studies on *Z. bungeanum* polyphenols to date. Notably, no severe adverse reactions associated with these polyphenols have been reported across these clinical investigations, providing a certain degree of evidence supporting their safety profile.

**Table 3:** Completed clinical trials utilizing *Z. bungeanum* polyphenols.

Compound	ID	Study phase	Dose & Administraton	Condition	Publication
Quercetin and Rutin	NCT01847521	2	Quercetin 70 mg/10kg/d and Rutin 30 mg/10kg/d po	Autism Spectrum Disorders	/
Rutin	NCT03437902	2 & 3	180mg po	Type 2 Diabetes Mellitus	/
Quercetin	NCT00913081	4	500-2000 mg po one time	Flushing	/
	NCT01708278	1	500-2000 mg/d po	Chronic Obstructive Pulmonary Disease	(118)
	NCT02463357	4	1000mg/d po	Mountain Sickness	/
	NCT01722669	1	500mg po	Healthy	(119)
Epicatechin	NCT01856868	1 & 2	100mg/day, po	Becker Muscular Dystrophy	/
	NCT03236662	2	100mg/day, po	Becker Muscular Dystrophy	/
	NCT01691404	/	100mg/d po	Hypertension, Endothelial Dysfunction	(120–122)
Naringenin	NCT01091077	1	1000mg po	HCV Infection	/
	NCT04697355	/	900mg/d po	Energy Expenditure Safety Issues Glucose Metabolism	/
Kaempferol	NCT06060691	1	Topical	Female Sexual Dysfunction	/
Arbutin	NCT03868748	1	150-400mg/d po	Healthy Volunteers	/
Chlorogenic acid	NCT02245204	1	Injection*	Advanced Cancer	/
	NCT02136342	1	Injection*	Advanced Cancer	/
	NCT02728349	1	Injection*	Glioblastoma	/
	NCT02728349	1	Injection*	Glioblastoma	/
	NCT03758014	2 & 3	Injection* 3 mg/kg/d	Glioblastoma	/
	NCT02621060	2	1200 mg/d po	Impaired Glucose Tolerance	/
	NCT02929901	2 & 3	200mg/d po	Type 2 Diabetes Nonalcoholic Fatty Liver	(123)

## 6 Inflammatory diseases and other compositions in *Z. bungeanum*

### 6.1 Alkaloids

Alkaloids, such as hydroxy-alpha-sanshool (HAS), constitute the characteristic compounds in *Z. bungeanum*, contributing to the notable sensation of numbness in the mouth (124). In a rat model of type 2 diabetes mellitus (T2DM), Zanthoxylum alkylamides (ZA), a mixed extract containing hydroxyl-γ-sanshool, hydroxyl-β-sanshool, and hydroxyl-α-sanshool, demonstrated the ability to control inflammation and address protein metabolism disorders, consequently ameliorating T2DM. The PI3K/Akt/forkhead box O signaling pathway and the TNFα/NF-κB pathway are implicated in this process (125).

Among the alkaloids in *Z. bungeanum*, HAS has been extensively studied for its anti-inflammatory effects. HAS exhibits a neuroprotective effect on H2O2-stimulated PC12 cells without inducing cytotoxicity in normal PC12 cells. The suppression of apoptosis is achieved by regulating the PI3K/Akt signaling pathway(126). Oral administration of HAS markedly improves spontaneous locomotion, cognitive function, and histopathological injuries in a mouse model of Alzheimer’s disease induced by D-galactose and AlCl3. The therapeutic effect of HAS involves the mitigation of oxidative stress damage and the activation of the Nrf2/HO-1 signaling pathway (127). As one of the main active ingredients in the herbal medicine TU-100, HAS enhances the production of antimicrobial defense molecules (ADM) by intestinal epithelial cells. TU-100, administered orally, prevents weight loss and colon ulceration in both TNBS-induced type-1 model colitis and OXN-induced type-2 model colitis. This suggests that HAS possesses anti-inflammatory properties and could potentially serve as a beneficial treatment agent for UC through the promotion of ADM production (128).

Zanthoxylin, another major alkaloid of *Z. bungeanum*, exhibits anti-inflammatory and pain-relieving effects in a variety of animal models. In mice, zanthoxylin alleviates pain in both general and formaldehyde-induced pain models. Its mechanism of action involves binding to the α7nAChR receptor and activating the JAK2/STAT3 signaling pathway, thereby inhibiting inflammation and reducing the production of pro-inflammatory cytokines such as IL-6 and TNFα (129).

### 6.2 Fatty acid

Research on the anti-inflammatory properties of fatty acids in *Z. bungeanum* predominantly focuses on *Z. bungeanum* seed oil (ZBSO). The primary components of ZBSO include eicosoic acid, linolenic acid, linoleic acid, oleic acid, palmitic acid, arachidonic acid, stearic acid, eicosenoic acid, and docosahexenoic acid (130). In LPS-triggered lung epithelial cells, ZBSO effectively inhibits the production of pro-inflammatory cytokines and chemokines, including IL-6, IL-10, TNFα, PGE2, MMP2, MMP9, MCP1, and COX2. This inhibition is achieved by blocking the TLR4/MyD88/NF-κB signaling pathway. Additionally, ZBSO inhibits the nuclear translocation of NF-κB/p65 (131).

*Zanthoxylum bungeanum* Maxim seed (ZBMS), rich in oleic acid, linoleic acid, and α-linolenic acid, exhibits potential for treating asthma and stress-related disorders (132). ZBMS protects mice from histamine/acetylcholine-induced asthma, reduces citric acid-induced cough in guinea pigs, and increases swimming endurance and survival time in mice, indicating a positive anti-stress effect. In an OVA-induced airway inflammation mouse model, ZBMS treatment improved lung peak inspiratory airflow in a dose-dependent manner (132).

Another group examined ZBSO in an OVA-induced asthmatic mouse model, demonstrating its efficacy in alleviating airway inflammation, attenuates lung tissue injury and airway remodeling, and inhibits leukocytes and eosinophils infiltration into the airway. ZBSO also reduces IL-5 and IL-4 in the bronchial airway, attenuates the induction of ICAM-1 and TNFα mRNA and protein expression levels, and alleviates ERK, JNK phosphorylation, c-fos and c-JUN induction in the lung tissue (133). ZBSO exhibits effective anti-inflammatory properties in the wound healing process. In SD rat models with deep second-degree burns, topical ZBSO application resulted in decreased levels of TNFα, IL-6, and IL-1β in serum, elevated IκBα, and reduced p-IκBα and p-NF-κB p65 expression (134). In copper comb-induced rat burn model, ZBSO can reduce the level of thiobarbituric acid reactant, IL-6, TNFα, increase GSH level and promote wound recovery (135). ZBSO also inhibits inflammation in bone-destroying diseases. In RAW264.7 cells stimulated with NF-κB ligand (RANKL), ZBSO decreases NF-κB, TNFα, NFATc1, and TRAP, leading to the inhibition of osteoclastogenesis. Among the fatty acids in ZBSO, alpha-linolenic acid (ALA) exhibits the strongest effect. In ovariectomized osteoporotic rats, preventive and therapeutic interventions with ALA resulted in decreased levels of IL-1β, IL-6, TAK1, TRAP, NFATc1, and TNFα (136).

### 6.3 *Z. bungeanum* essential oil

*Z. bungeanum* essential oil (ZBEO) is the primary source of the distinctive flavor of Sichuan pepper, with terpenoids being a major component of ZBEO (2). ZBEO has demonstrated anti-inflammatory effects in various skin disease models. In a guinea pig model of psoriasis, ZBEO treatment significantly improved Baker scores and reduced inflammatory cell infiltration (137). In a mouse model of ultraviolet-induced skin photoaging, topical application of ZBEO improved photoaging damage, reduced skin thickening, and attenuated inflammatory cell infiltration. ZBEO also inhibits the levels of MMP9, MMP1, and MMP3 in skin tissue, enhance the activity of CAT, SOD, and GSH-Px/GPX, and reduced the production of the lipid peroxidation byproduct MDA. Furthermore, ZBEO effectively suppress the expression of TNFα, IL-6, IL-1β, and IL-1α (138). In a HaCaT cell inflammatory model induced by Propionibacterium acnes (P. acnes), pretreatment with ZBEO reduced the levels of TNFα, IL-1β, IL-8, and IL-6, as well as the mRNA levels of TLR2, IL-8, IL-6, and NF-κB (139).

ZBEO also shows therapeutic effects in gastrointestinal disorders due to its anti-inflammatory properties. ZBEO has demonstrated protective effects against DSS-induced colitis in mice. ZBEO doses of 20-80 mg/kg reduced myeloperoxidase activity, colonic pathological damage, colon length shortening, disease activity index, and DSS-induced weight loss (140, 141). Administration of ZBEO significantly reduced IL-1β, IL-12 (140), TNFα, VCAM-1, TLR8, and IL-11 (141) mRNA levels. ZBEO is reported to inhibit inflammation in colitis in mice by regulating the PPARγ and NF-κB pathways, and suppressing NLRP3 activation (140). Next-generation sequencing (NGS) verifies that ZBEO increases VCAM-1 and CYP, and suppresses CXCL and S100A8 to attenuate UC symptoms (141). *In vitro* studies also demonstrate that ZBEO can reverse the imbalanced expression of IL-1β, IL-6, IL-10, and TNFα in LPS-induced NCM460 colon epithelial cells (141).

ZBEO inhibited enteritis and intestinal dysfunction caused by E. coli infection in mice. Histopathological observations indicated that ZBEO significantly improved the impairment of intestinal tissue structure, which could be associated with its inhibitory effect on the gene expression of inflammatory cytokines such as IL-8, TNFα, TLR4, and TLR2 (142). Atomized inhalation of ZBEO protects mouse from inflammation related colorectal cancer by reducing inflammation and cancer transformation. Furthermore, a decrease in AChE activity, an increase in ChAT activity, an increase in α7nAChR expression, and a decrease in IL-6 mRNA levels are observed in ZBEO treated group (143).

### 6.4 Other extractions

*Z. bungeanum*-cake-separated moxibustion (ZBCS-moxi) is a traditional Chinese therapy that has been employed for centuries to treat rheumatoid arthritis. A recent study assessed the anti-inflammatory effects of ZBCS-moxi in a rat model of rheumatoid arthritis. The study found that rats treated with ZBCS-moxi for three weeks exhibited a significant reduction in paw volume, pannus formation, synovial hyperplasia of synovial membranes, and levels of TNFα and IL-1β in serum (144).

These findings suggest that Zanthoxylin and ZBCS-moxi may have therapeutic potential for the treatment of inflammation and pain. However, more research is needed to confirm these findings through clinical trials.

## 7 Discussion

*Zanthoxylum bungeanum Maxim*., or Chinese prickly ash, holds a rich history spanning over two millennia in traditional Chinese medicine (5). This herb has been extensively used orally and topically to address various ailments, including gastrointestinal discomfort, arthritis, and bruises (5, 7). Its significance extends beyond China, finding a place in traditional medical practices in countries such as India and Nepal (7). Additionally, the unique flavor and numbing taste of the dried fruit follicles of *Z. bungeanum* have made it a significant ingredient in Chinese cuisine (1). Over time, research on and applications of *Z. bungeanum* have expanded significantly. *Z. bungeanum* exhibits diverse pharmacological activities such as anti-inflammatory, analgesic, antibacterial, and anti-tumor properties, showcasing therapeutic effects on multiple organ systems, including the gastrointestinal tract, cardiovascular system, and nervous system (2). The plant contains over 140 compounds, including polyphenols, alkaloids, lignans, coumarin, fatty acids, and essential oils (2, 11, 12). Beyond its polyphenolic content, constituents like hydroxy-alpha-sanshool, a mixed extract of fatty acids, and essential oil extraction from *Z. bungeanum*, have proven anti-inflammatory efficacious in various systems, such as the nervous system (127) and digestive system (140, 141). As research progresses, the application of *Z. bungeanum* in both medical and daily contexts continues to broaden, promising potential benefits to human health.

Polyphenols from *Z. bungeanum* emerge as a promising class of natural compounds with potential health benefits, particularly in preventing and treating inflammatory diseases. Numerous studies have highlighted their anti-inflammatory and antioxidant properties through a variety of mechanisms, including:

- Inhibiting pro-inflammatory cytokine production, such as IL-1β, TNFα, and IL-6.
- Suppressing the NF-κB and MAPK signaling pathway, central to inflammation.
- Activating the Nrf2/HO-1 signaling pathway, protecting cells from oxidative damage.
- Modulating the immune response, promoting regulatory T cells and suppressing inflammatory T cells.

While much of the current research on polyphenols of *Z. bungeanum* has been conducted *in vitro* or in animal models, promising preclinical data suggest therapeutic potential for a range of inflammatory diseases in humans, including ulcerative colitis, arthritis, asthma, chronic obstructive pulmonary disease, cardiovascular disease, and neurodegenerative diseases. In addition to their anti-inflammatory effects, polyphenols of *Z. bungeanum* have demonstrated other beneficial properties, such as anti-cancer, anti-diabetic, anti-bacterial, and neuroprotective effects.

Several clinical trials have tested *Z. bungeanum* polyphenols in non- inflammatory diseases, indicating the safety of these compound. Future research should prioritize human clinical trials to validate the clinical efficacy of polyphenols of *Z. bungeanum* on inflammatory diseases. Additionally, researchers should investigate:

- Optimal dosages and long-term safety of polyphenols of *Z. bungeanum*.
- Synergistic or antagonistic interactions of polyphenols of *Z. bungeanum* with other bioactive substances.
- Effects of polyphenols of *Z. bungeanum* on specific biomarkers of inflammation and disease activity.
- Mechanisms by which polyphenols of *Z. bungeanum* exert their beneficial effects.

Ultimately, research outcomes may contribute to the development of novel therapeutic interventions and dietary recommendations that harness the power of polyphenols of *Z. bungeanum* to improve human health and well-being. For example, polyphenols of *Z. bungeanum* could be used to develop:

- New drugs or dietary supplements for the prevention and treatment of inflammatory diseases.
- Functional foods or fortified beverages that promote overall health and well-being.
- Personalized nutrition plans that take into account individual genetic and environmental risk factors.

Overall, the polyphenols of *Z. bungeanum* are a promising class of natural compounds with the potential to play a significant role in human health and well-being. Further research is needed to fully elucidate their mechanisms of action and develop safe and effective therapies for human use.

## Author contributions

GH: Funding acquisition, Investigation, Writing – review & editing. JQ: Conceptualization, Data curation, Writing – original draft. ZP: Validation, Visualization, Writing – original draft. XW: Resources, Validation, Visualization, Writing – original draft. NZ: Visualization, Writing – review & editing. XJ: Conceptualization, Funding acquisition, Investigation, Project administration, Resources, Writing – review & editing.
